# Patellar tendon ossification after partial patellectomy: a case report

**DOI:** 10.1186/1752-1947-4-47

**Published:** 2010-02-09

**Authors:** Husamettin Cakici, Onur Hapa, Kutay Ozturan, Melih Guven, Istemi Yucel

**Affiliations:** 1 Department of Orthopaedic Surgery, Abant Izzet Baysal University Hospital. Bolu, Turkey; 2 Department of Orthopaedic Surgery, Izzet Baysal State Hospital, Bolu, Turkey; 3 Department of Orthopaedic Surgery, Duzce University Hospital, Duzce, Turkey

## Abstract

**Introduction:**

Patellar tendon ossification is a rare pathology that may be seen as a complication after sleeve fractures of the tibial tuberosity, total patellectomy during arthroplasty, intramedullary nailing of tibial fractures, anterior cruciate ligament reconstruction with patellar tendon autograft and knee injury without fracture. However, its occurrence after partial patellectomy surgery has never been reported in the literature.

**Case presentation:**

We present the case of a 35-year-old Turkish man with a comminuted inferior patellar pole fracture that was treated with partial patellectomy. During the follow-up period, his patellar tendon healed with ossification and then ruptured from the inferior attachment to the tibial tubercle. The ossification was excised and the tendon was subsequently repaired.

**Conclusion:**

To the best of our knowledge, this is the first report of patellar tendon ossification occurring after partial patellectomy. Orthopaedic surgeons are thus cautioned to be conscious of this rare complication after partial patellectomy.

## Introduction

Patellar tendon ossification is a rare occurrence. Whenever reported, it is usually associated with conditions such as conservatively treated sleeve fractures of tibial tuberosity [1], total patellectomy during arthroplasty [2], intramedullary nailing of tibial fractures [3], anterior cruciate ligament reconstruction with patellar tendon autograft [4], and knee injury without fracture [5].

We report here a case of comminuted displaced inferior pole fracture of the patella that was treated with partial patellectomy. During the follow-up period the patellar tendon healed with ossification. To the best of our knowledge, this is the first reported clinical case of patellar tendon ossification occurring after partial patellectomy. The purpose of this report is to point out this rare complication of patellar tendon rupture.

## Case presentation

**Figure 1:**
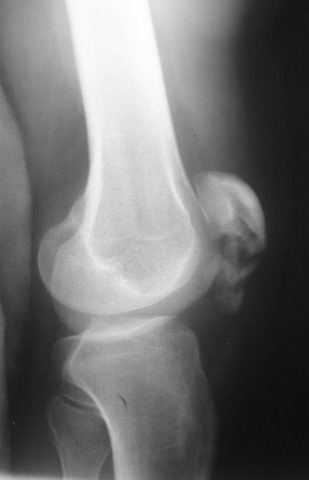
**Preoperative radiograph of the patient**.

**Figure 2:**
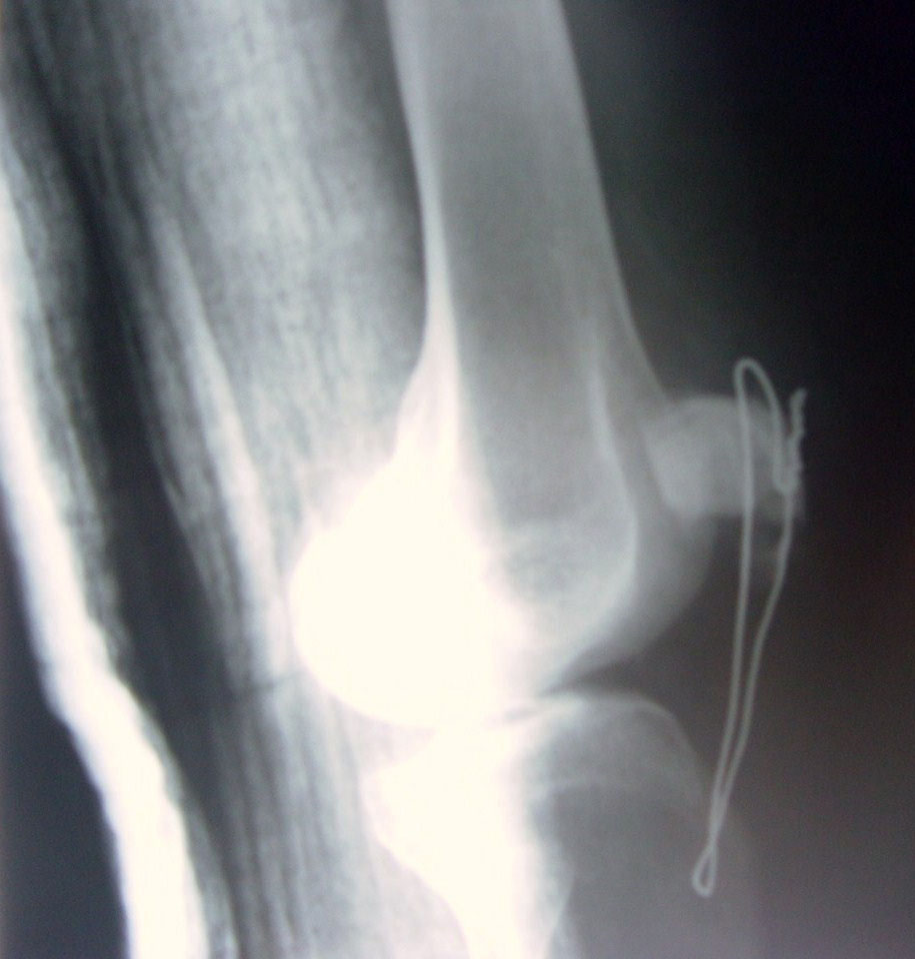
**Postoperative lateral radiograph of the patient**.

A 35-year-old Turkish man who had fallen on his flexed right knee while walking on ice was referred to our hospital. He suffered from pain and inability to move his right knee. Physical examination revealed prominent swelling and tenderness over the right patella. Plain radiographs showed comminuted displaced inferior pole fracture of the patella (Figure 1). His extremity was immobilized initially through a cast brace, and he was then operated under general anesthesia on the following day. During the operation, it was found out that the patellar fracture could not be reduced and repaired. Because of this, a partial patellectomy was performed and his patellar tendon was sutured to the patella with No. 2 polydioxanone (PDS) sutures and augmented with a cerclage wire (Figure 2). A long leg cast was then applied and we advised our patient to move using two crutches and bear no weight for three weeks. At the end of the third week, he was started on active and passive ranges of motion exercises of the knee.

**Figure 3:**
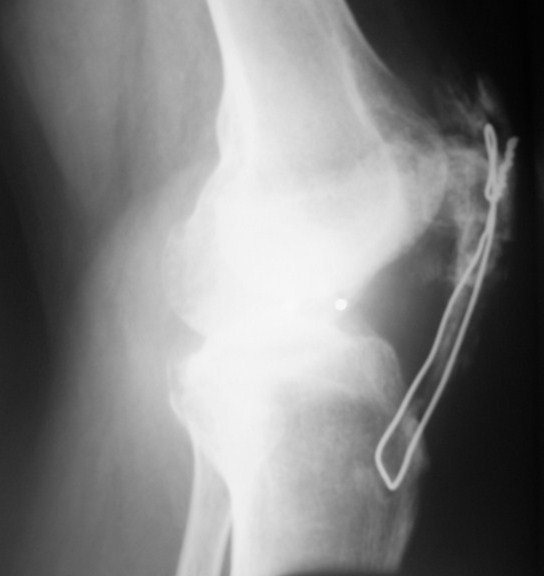
**Extensive heterotopic ossification of the patellar tendon**.

On his follow-up visit after six weeks, he was already able to flex his right knee by about 100°. After that period, however, he started to experience a gradual decrease in knee movement. Two months after the operation, the active flexion of his knee was only 60°. A lateral radiograph of his right knee showed extensive ossifications at the resected part of the patella and calcifications in the patellar tendon (Figure 3). Excision of the ossifications and implant removal were thus planned. During that period, he felt a sudden pain over the tibial tubercle of his right leg while he was descending the stairs. He was unable to extend his right knee.

**Figure 4:**
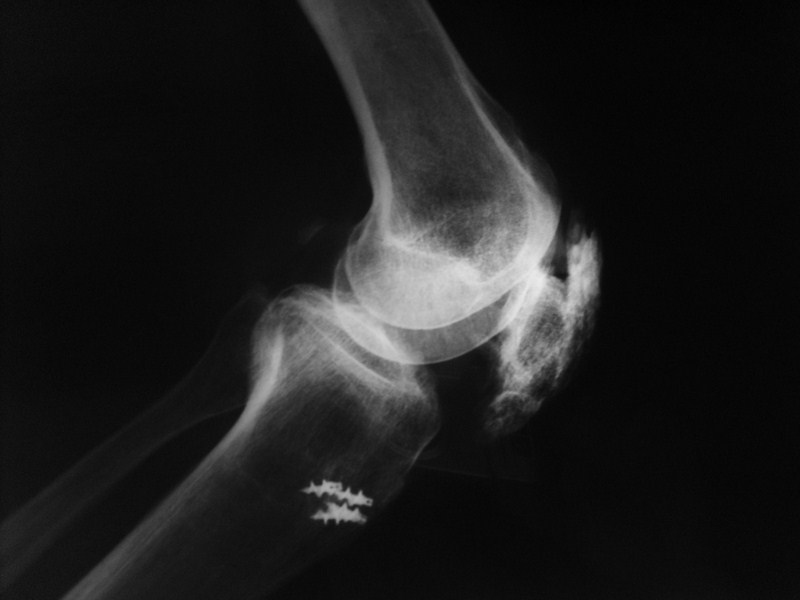
**Postoperative lateral radiograph of the patient**.

**Figure 5:**
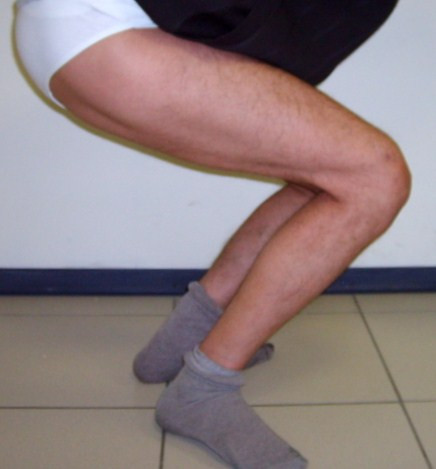
**Clinical picture of the patient five years after the operation showing the degree of active knee flexion**.

Plain radiographs revealed the presence of patella alta. As a result, another operation was performed. It was then discovered that the patellar tendon with ossification throughout its length was avulsed from the tibial tubercle. The cerclage wire from the previous surgery was removed and ossifications were excised. Patellar tendon was fixed to the tibial tubercle with four suture anchors (Figure 4). After three weeks of knee immobilization with a long leg cast, a gradually increasing range of knee motion rehabilitation was applied. Full weight-bearing was allowed after six weeks. At the end of the fifth postoperative year, the range of motion of his right knee was 90° flexion and full extension without any pain (Figure 5).

## Discussion

The ideal treatment of inferior pole fractures of the patella remains a controversial issue. The options include internal fixation of the pole fragment and resection of the avulsed fragment with repair of the patellar tendon to the patella [6]. In experimental studies [7,8], enlargement of the remaining patella and patellar tendon calcification after partial patellectomy were demonstrated in rabbits at about 24 weeks after surgeries were performed on them.

However, in clinical studies where the results of partial patellectomy were reported, incidences of extensive patellar tendon ossification were not detected [9,10]. Saltzman *et al*. [9] reported that patellar length and the area of retained fragment were found to be enlarged in varying degrees in some patients. They concluded that this was different from the calcification or ossification phenomenon that could be seen at the extensor mechanism after a total patellectomy or the development of an osseous spur where the patellar tendon was reattached.

In our patient, the tendon was ruptured neither from the repaired bone-tendon junction nor throughout the length of the ossified tendon, but rather from an unexpected part, which is the tibial tubercle. An explanation for this may be the contraction of the quadriceps muscle that led to the rupture at the distal, weaker, nonossified ligamentous part of the ossified tendon. Cerclage wire might put additional pressure on the distal attachment of the tendon that was supplementing the rupture.

The only other report of ossified patellar tendon rupture to be found in the literature was by Yoon *et al*. [11] who described a case in which the ossified tendon ruptured in a z-like fashion from the proximal medial aspect to the distal lateral aspect. This differs from our patient's condition in that he had a prior partial patellectomy and the ossified tendon avulsed completely from its insertion into the tibial tubercle alone.

## Conclusion

Surgeons must be cautious about patellar tendon ossification after partial patellectomy because this can lead to patellar tendon rupture.

## Competing interests

The authors declare that they have no competing interests.

## Consent

Written informed consent was obtained from the patient for publication of this case report and any accompanying images. A copy of the written consent is available for review by the Editor-in-Chief of this journal.

## Authors' contributions

HC and OH contributed to this case report's conception and design. They also performed the literature research, prepared the manuscript and reviewed it for publication. KO, MG and IY were involved in the literature review and helped draft parts of the manuscript. HC supervised the writing of the manuscript. HC and IY supervised the general management and follow-up of the patient. All authors have read and approved the final manuscript.
